# Fatima Suleman: making medicines more widely available

**DOI:** 10.2471/BLT.17.031217

**Published:** 2017-12-01

## Abstract

The availability of affordable medicines is a major barrier to providing high-quality health care in many countries. Fatima Suleman talks to Fiona Fleck.

Q: What was your experience of growing up in South Africa in the 1970s? What motivated you to work on making medicines affordable?

A: I grew up in the apartheid era and, as an Indian, I and other members of our group faced discrimination. My schooling was in an Indian-only school, my university education was at a so-called Indian university, where the choice of subjects was limited, and there were areas where I was not allowed to go. Many students were involved in the freedom struggle and that inspired me to strive for equity and justice. I was also influenced by the Islamic Medical Association that provided health care via mobile and fixed clinics run by nurses. I volunteered to work with the association in rural, deprived areas of South Africa, where we saw patients queue up in the hope of receiving health care. There was such disparity between the haves and have-nots. Today, young South Africans take access to education and health services for granted.

Q: Under apartheid, inequities were anchored in the law. How was this reflected in access to medicines before the transition to democracy in 1994?

A: Before 1994, health services in South Africa were fragmented, as dictated by the apartheid policy of racial segregation. However, the provision of separate services for different racial groups predates the apartheid laws and goes back to the Public Health Amendment Act of 1897. Under apartheid, there were 14 different health departments providing mainly curative services through hospitals and the pharmaceutical supply system was fragmented as well. There were separate systems for selecting medicines for curative services and preventive services. Each bantustan – partially self-governing area for indigenous people during apartheid – operated its own medicines selection process and these processes were biased by individual prescribers.

Q: Can you tell us about your first work experiences as a pharmacist?

A: I worked at the overcrowded and overstretched King Edward VIII Hospital in Durban with a strong team of pharmacists who practised evidence-based medicine. I began to understand why pharmacists need to manage the use of medicines to ensure their rational use and to stand firm against physicians’ pressure to prescribe irrationally. I could see how expensive medicines were a drain on health budgets. Also, around this time, the South African National Drug Policy was developed, reflecting – for the first time in a policy document – the need to provide equitable access and affordable medicines for all.

Q: How did you start making medicines more accessible in your country? How did activists, such as Zackie Ahmat from the Treatment Action Campaign (TAC), contribute?

A: I was involved in the review of the Primary Health Care Standard Treatment Guidelines and Essential Medicines List in 1998. We discussed what constitutes equitable and affordable health care. My postgraduate studies looked into problems with prescribing, including prescription of different medicines for patients with the same diagnoses, and unnecessary prescription of new and expensive medicines. There was a real need to promote generic use and affordable medicines. The TAC and other activists helped bring the high price of medicines to the fore in the 1990s and early 2000s. They also fought the stigma attached to HIV, and campaigned to increase access to antiretrovirals and other medicines. We have all benefited from their efforts.

“Over the last 20 years, South Africa has developed a strong system for reviewing and implementing the national essential medicines programme.”

Q: What are the challenges for making essential medicines more available in South Africa and other developing countries?

A: Over the last 20 years, South Africa has developed a strong system for reviewing and implementing the national essential medicines programme. Prescribing decisions are more transparent than they used to be and we have standard treatment guidelines for different levels of care. These guidelines have mostly been incorporated into the undergraduate health sciences curriculum, so that many practitioners do not remember a time without such guidelines. There are countries in Africa where health workers still don’t understand the benefits of taking an evidence-based approach to medicine selection, supply and use. Health workers need to apply this approach to the entire health system and not just at primary care level. Some specialists think that the lists do not apply to them and that they should be allowed to prescribe whatever they like. Some countries in Africa that I visited recently also face this challenge and are striving to promote a better understanding of why specialists should use guidelines on essential medicines.

Q: What are the challenges today for countries that recognize the benefits of evidence-based management of medicines?

A: One challenge is that high-priced medicines are appearing on the World Health Organization (WHO) Model Lists of Essential Medicines. Many developing countries cannot afford to put these on their own national lists. We need to explain that these recommendations are based on good evidence, but should only be adopted by countries that can afford them. Another challenge is that many countries – including South Africa – have yet to fully incorporate their national essential medicines lists in their undergraduate health sciences curricula. Future prescribers’ training must be based on these guidelines.

Q: Which essential medicines are too expensive for your country?

A: Some cancer medicines and hepatitis C medicines are too expensive for our country. Some of these appear on the WHO list, but not the South African essential medicines list. Some second-line antiretrovirals and medicines for multidrug-resistant and extensively drug resistant tuberculosis are included on our list – even though they are very expensive – and so our health ministry is trying to negotiate lower prices. New vaccines are another one of the biggest expenditure items. As chair of the pricing committee in South Africa, I must balance industry interests, sustainability and patients’ interests in the private sector.

Q: What can governments do to reduce these prices?

A: WHO outlines the various policies to reduce medicine prices in the *WHO guideline on country pharmaceutical policies*. I was on the expert panel that provided input on these guidelines. The policies options include the regulation of price mark ups, tax exemptions for pharmaceutical products, the use of health technology assessment and the promotion of generic medicines. Also, Zaheer Babar from Huddersfield University published a book in 2015 on pricing policies in different countries that describes what has been implemented to date and the impact of these policies.

“We need to remind the industry that R&D models are still evolving and R&D is needed in neglected areas, such as tuberculosis, malaria and antibiotics.”

Q: Can you give examples of how some developing countries are increasing access to medicines?

A: Several countries can combine their regulatory activities. For example, the East African Community Medicines Regulatory Harmonization and the Zazibona initiative in Botswana, Namibia, Zambia and Zimbabwe pool scarce skills to undertake medicine reviews for registration and inspections of manufacturing facilities. The high cost of medicines is a major issue. Health ministers in Africa meet regularly to discuss pricing and to find ways to harmonize their regulatory activities for medicines. WHO’s regional office for Africa is also helping to promote a better understanding of the need for evidence-based medicines policies.

Q: Can you tell us about your work in other countries?

A: I helped to review the medicines policies and reduce medicine prices in Malaysia and Ethiopia. I worked in [the Republic of] Tanzania to strengthen evidence-based use of essential medicines. At the Faculty of Science at Utrecht University I worked on approaches to increasing access to medicines. Today many people are committed to making medicines affordable and it’s encouraging to see this movement gaining momentum.

Q: The pharmaceutical industry says it cannot reduce the prices of new products because of the high cost of research and development (R&D). Critics say the industry is exaggerating these costs. What does R&D really cost?

A: We don’t know the real R&D costs. A number of studies provide for theoretical costs, but these estimates need further debate. The question is do we just stick to a business- and patent-heavy model, or do we seek a more public health-oriented type of model?

Q: What are the prospects of success for a project such as the Médecins Sans Frontières-initiated Life Prize (formerly the 3P project) that aims to stimulate tuberculosis drug development with the award of a cash prize?

A: There are several projects underway to stimulate innovation for medicines, diagnostics and vaccines beyond the patent-based R&D system. This is a good idea because we need to remind the industry that R&D models are still evolving and R&D is needed in neglected areas, such as tuberculosis, malaria and antibiotics.

Q: Can the generics industry make essential drugs and medicines more widely available?

A: The generics industry can be a means of providing affordable access to medicines, but the industry needs to be monitored and, if necessary, regulated to ensure that medicine prices are not too high and that monopolies are not introduced. Countries should take care not to introduce regulations and policies that might conflict with one another and thereby prevent maximum use of generics in countries.

Q: Should countries make better use of the exceptions in the Agreement on Trade-Related Aspects of Intellectual Property Rights, an international legal agreement between members of the World Trade Organization?

A: This option should be considered when developing countries face pressure to introduce new expensive essential medicines. Pharmaceutical company Gilead recently agreed to include four middle-income countries – Belarus, Malaysia, Thailand and Ukraine – in a voluntary licence for generic drug sofosbuvir for hepatitis C. The company had been under increasing pressure from these countries. Compulsory licenses should be a last resort. Countries should first try to negotiate prices with the pharmaceutical industry.

